# Mesenchymal Cells Affect Salivary Epithelial Cell Morphology on PGS/PLGA Core/Shell Nanofibers

**DOI:** 10.3390/ijms19041031

**Published:** 2018-03-29

**Authors:** Lauren Sfakis, Tim Kamaldinov, Alexander Khmaladze, Zeinab F. Hosseini, Deirdre A. Nelson, Melinda Larsen, James Castracane

**Affiliations:** 1 Colleges of Nanoscale Science and Engineering, SUNY Polytechnic Institute, Albany, NY 12203, USA; LSfakis@sunypoly.edu (L.S.); TKamaldinov@sunypoly.edu (T.K.); 2 Department of Physics, University at Albany, State University of New York, Albany, NY 12222, USA; 3 Department of Biological Sciences, University at Albany, State University of New York, Albany, NY 12222, USA; zfarajallahhosseini@albany.edu (Z.F.H.); dnelson@albany.edu (D.A.N.)

**Keywords:** core/shell nanofibers, co-culture, tight junctions, poly glycerol sebacate, salivary tissue engineering

## Abstract

Engineering salivary glands is of interest due to the damaging effects of radiation therapy and the autoimmune disease Sjögren’s syndrome on salivary gland function. One of the current problems in tissue engineering is that in vitro studies often fail to predict in vivo regeneration due to failure of cells to interact with scaffolds and of the single cell types that are typically used for these studies. Although poly (lactic co glycolic acid) (PLGA) nanofiber scaffolds have been used for in vitro growth of epithelial cells, PLGA has low compliance and cells do not penetrate the scaffolds. Using a core-shell electrospinning technique, we incorporated poly (glycerol sebacate) (PGS) into PLGA scaffolds to increase the compliance and decrease hydrophobicity. PGS/PLGA scaffolds promoted epithelial cell penetration into the scaffold and apical localization of tight junction proteins, which is necessary for epithelial cell function. Additionally, co-culture of the salivary epithelial cells with NIH3T3 mesenchymal cells on PGS/PLGA scaffolds facilitated epithelial tissue reorganization and apical localization of tight junction proteins significantly more than in the absence of the mesenchyme. These data demonstrate the applicability of PGS/PLGA nanofibers for epithelial cell self-organization and facilitation of co-culture cell interactions that promote tissue self-organization in vitro.

## 1. Introduction

Salivary hypofunction is a deleterious condition often resulting from the autoimmune disease Sjögren’s syndrome or as a side-effect in patients who receive radiation therapy for head and neck tumors, and other causes. Salivary glands are highly branched organs that produce and secrete saliva to aid in digestion of food, lubrication, and oral cavity homeostasis. Millions of individuals suffer from salivary gland hypofunction, leading to xerostomia and other issues that decrease quality of life. Current treatments for this disease are inadequate and cause unwanted side effects [1]. Therefore, new therapeutics such as tissue engineering are necessary to restore function to damaged glands. 

Nanofibers have been widely used in tissue engineering: they provide scaffolds with increased surface area, allowing for the organization of cells into a monolayer and resemble the native extracellular matrix (ECM) and basement membrane that are critical for gland development and regeneration [2,3,4]. Nanofiber scaffolds promote apico-basal polarity of salivary epithelial cells, a critical cell property for functional secretion. A common polymer used in engineering of both soft and hard tissues is poly-lactic-co-glycolic acid (PLGA) (stiffness ~ 20 MPa–2.0 GPa) [5,6]. PLGA nanofibers are currently in use for bone regeneration [7], wound healing [8,9], and drug delivery [10]. PLGA has also been demonstrated to be useful in salivary gland regeneration in vitro. Cantara et al. demonstrated the use of functionalized PLGA nanofibers with laminin-111 in the promotion of apical localization of the tight junction protein, occludin [11]. Tight junctions are protein complexes that form junctions between epithelial cells that assemble towards the apical surface to control paracellular movement of ions and molecules between cells [12]. Soscia et al. used photolithographic techniques to demonstrate that hemispherical micropatterned arrays that mimic the dimensions of salivary secretory acinar structures manipulate epithelial cell shape and induce subcellular organization; cells in appropriately sized “craters” showed increased monolayer cell heights and expression and apical localization of the tight junction protein, occludin, relative to larger diameter craters or flat surfaces [13]. Given these in vitro studies and the known fast resorption of PLGA in vivo, PLGA nanofiber scaffolds hold promise for application in salivary gland regenerative strategies. 

In recent years, it has become increasingly clear that scaffold mechanical properties are critical for soft tissue engineering applications [14,15,16]. Tissues are known to have viscoelastic properties, namely properties of elastic solids and viscous liquids. Peters et al. summarized the importance of scaffold stiffness on salivary gland development by varying the mechanical properties of polyacrylamide gels [17]. It was concluded that mechanical properties in the range of the tissue in vivo plays a large role in in vitro gland regeneration [17]. Atomic Force Microscopy (AFM) was used to measure the bulk compliance properties of adult submandibular salivary glands, which was found to range from 1.66 ± 0.44 kPa to 3.05 ± 0.88 kPa [18]. Despite the positive response of salivary epithelial cells to PLGA nanofibers, the low compliance of PLGA is likely to be less than ideal for promotion of salivary gland regeneration. 

Poly (glycerol sebacate) (PGS) has been proposed for soft tissue engineering applications. It is more compliant than PLGA (stiffness ~ 0.056–1.5 MPa) [19] with a controlled linear surface degradation profile [20]. Applications include adipose [21], nerve [22], ocular [23] and myocardial tissue engineering [24]; however, no studies have used PGS in salivary gland engineering. PGS rapidly degrades into glycerol and sebacic acid, naturally existing metabolites in the body [25]. Electrospinning the material before crosslinking, due to its characteristic low molecular weight and low viscosity in many solvents, makes it hard to get it into a fiber form. After thermal crosslinking, this polymer becomes insoluble in many organic solvents needed for electrospinning. A useful technique to overcome these challenges and integrate PGS with PLGA nanofiber scaffolds is to incorporate this softer polymeric material through a core/shell electrospinning technique.

Core/shell electrospinning is a technique which has been emerging in recent years to modify and enhance the functionality of nanofiber structures. Two dissimilar polymer solutions are independently drawn through a co-axial spinneret capillary which are then spun to generate nanofibers with a core of one material and the sheath of another [26]. This method gives the versatility of incorporating two materials with different properties into one. Core/shell electrospinning is typically used to recover the electrospinning ability of polymers that are unable to form fibers themselves as well as improving the mechanical properties of the overall fiber system [27,28]. 

Soft tissue organs are composed of multiple dynamic cell types that interact with their immediate microenvironment, being able to sense up to the length of a neighboring cell body away [29]. In response to changes in the environmental stimulus, cellular functions are altered, such as growth, differentiation, adhesion, and survival [30]. Providing epithelium with a proper environmental stimulus can potentially recapitulate the in vivo environment and further improve in vitro epithelial development. Epithelial-mesenchymal interactions are known to be required for effective development [4] and these interactions are increasingly understood to be important for regeneration [31,32]. 

Here, we generated PGS/PLGA nanofiber scaffolds by core/shell electrospinning and used them for in vitro co-culture of salivary epithelial and mesenchymal model cell lines to recapitulate epithelial-mesenchymal interactions in vitro that may facilitate tissue organization and regeneration. The fabrication of core/shell nanofiber structure scaffolds was used to fine tune the mechanical properties of PLGA nanofibers without modifying the chemical environment. We used PGS/PLGA core/shell nanofibers and epithelial/mesenchymal co-cultures in this study to determine if “softer” scaffolds and cell co-culturing would affect the morphology and tight junction localization of salivary gland epithelial cells.

## 2. Results

### 2.1. Development and Characterization of PGS/PLGA Core/Shell Nanofibers

#### 2.1.1. Electrospinning Parameter Optimization and SEM Fiber Morphology 

**Figure 1:**
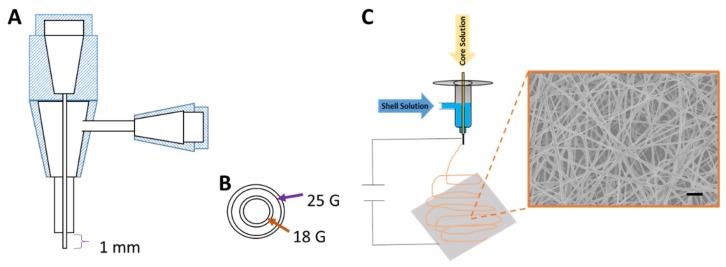
Schematic showing core/shell electrospinning spinneret. (**A**) Side view of spinneret, (**B**) Top-down of co-axial needles. Inner gauge size (orange) and outer gauge size (purple), (**C**) Electrospinning setup with SEM image of resulting core/shell nanofibers. Scale, 2 μm.

**Figure 2:**
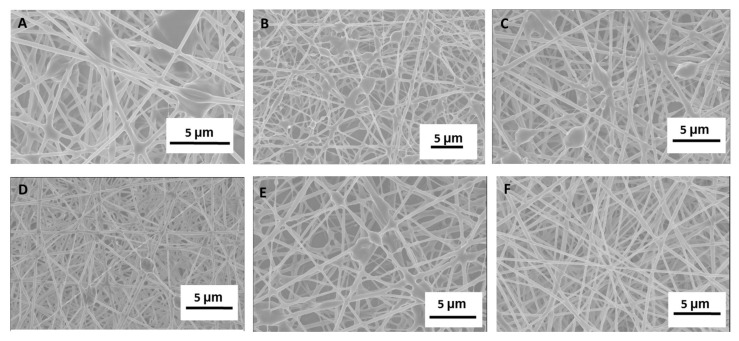
Optimization of core/shell electrospinning. Configurations investigated were: core flow rate (μL/min)/shell flow rate (μL/min)/voltage (kV)/distance (cm). SEM images of nanofibers formed under specific conditions: (**A**) 9/1.5/13/15, (**B**) 4.5/1.5/11/15, (**C**) 4/2/11/15, (**D**) 3/3/12.5/15, (**E**) 2.25/0.75/11/15, (**F**) 1.5/1.5/11/15. Configuration (**F**) showed uniform fiber mats that were comparable to PLGA fiber mats and was used throughout. Scale, 5 μm.

PGS/PLGA Core/Shell nanofibers were electro spun using a co-axial needle (Figure 1A,B) to produce an optimized fiber mat similar to our previously published 250 ± 50 nm PLGA nanofibers [33] while still incorporating as much PGS pre-polymer in the fiber strands as possible. Optimization of PGS/PLGA core/shell fiber mats was performed by changing the electrospinning parameters: core flow rates (μL/min), shell flow rates (μL/min), voltage (kV) and distance (cm), respectively. The voltage was changed to maintain a stable Taylor cone during fabrication, given stability issues with higher flow rates for the core solution. Characterization of fibers in which the parameters were varied was done by SEM. The 9/1.5/13/15 parameters showed an unstable Taylor cone and resulted in broken fibers with PGS-rich beads (Figure 2A). As the amount of PGS to PLGA flow rates decreased, unstable Taylor cones were still seen while spinning. Broken non-uniform fiber mats were observed under SEM (Figure 2B–E). The fiber mat with the electrospinning parameters 1.5/1.5/11/15 showed a homogenous fiber morphology and maintained a consistent stable Taylor cone during fabrication (Figure 2F). 

**Figure 3:**
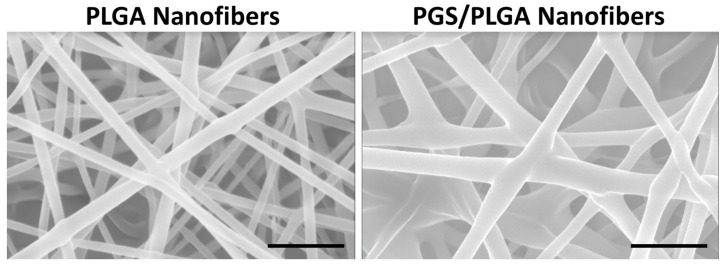
Comparison of fiber diameters for poly (lactic co glycolic acid) PLGA and poly (glycerol sebacate) (PGS)/PLGA core-shell nanofibers. SEM images. PLGA and PGS/PLGA fibers mats have average diameters of 190 ± 33 nm and 245 ± 61 nm, respectively, which was measured from SEM images. Scale, 1000 nm.

The optimized PGS/PLGA fiber mat using electrospinning parameters 1.5/1.5/11/15 was used throughout this study for further characterization. Comparing the PGS/PLGA fiber diameters to our original proposed PLGA nanofibers was performed taking 100 fiber diameters of three separate fiber mats. PGS/PLGA fiber mats showed slightly thicker fiber diameters than the PLGA nanofibers, 245 ± 61 nm and 190 ± 33 nm, respectively (Figure 3). Although we previously determined that salivary epithelial cells assume more epithelial-like morphology on nanofibers having diameters in the 200–250 nm range rather than on microfibers (>1 μm diameter) [34], the cells did not respond any differently to nanofibers in the 100 nm range [35], and so this small difference in diameter between the PGS/PLGA and PLGA is unlikely to a significant effect on the cells. 

#### 2.1.2. Physical Properties of PGS/PLGA and PLGA Fiber Mats

To determine the orientation of PGS and PLGA within the nanofibers, we developed a method to investigate this using Raman imaging [36]. Through confocal Raman spectroscopy of single fiber strands, it was previously confirmed that the 1.5/1.5/11/15 core/shell electrospinning configuration obtained specific chemical composition signatures of PGS in the core and PLGA in the shell [36]. 

To further characterize the fiber properties, we examined the surface wetness and compliance of both fiber mats. The PGS/PLGA core/shell nanofibers have a water contact angle of 65 ± 5 compared to PLGA 122 ± 4. Using AFM, a Young’s modulus of 1.3 ± 0.66 MPa and 4.8 ± 1.5 MPa was measured for PGS/PLGA and PLGA fiber mats, respectively (Table 1).

### 2.2. SIMS Cell Growth on PGS/PLGA Nanofiber Substrates

**Figure 4:**
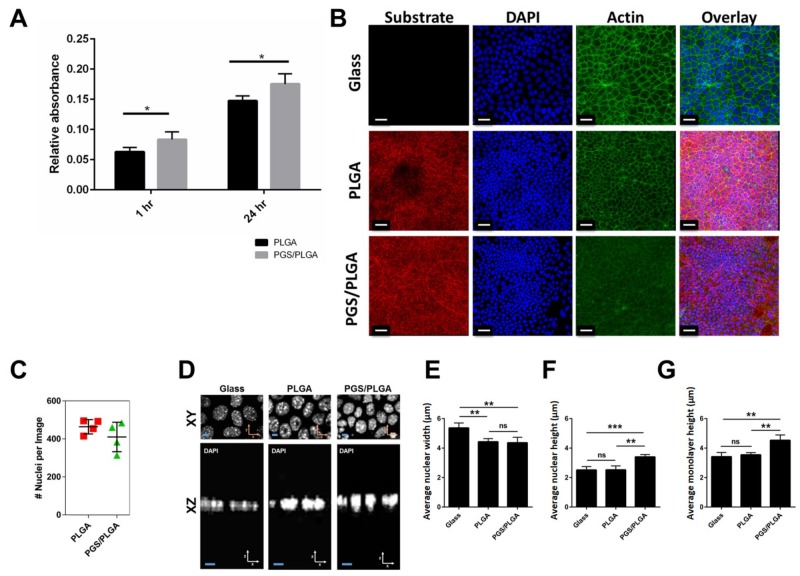
Softer PGS/PLGA scaffolds affect SIMS cell morphology. (**A**) MTT assay showing SIMS cells on PLGA and PGS/PLGA nanofiber scaffolds. Data are means ± SD (*n* = 5) * *p* < 0.05 unpaired *t*-test, (**B**) Maximum projection images of SIMS cell morphology following culture on Glass, PLGA or PGS/PLGA scaffolds (red) for 7 days. ICC: actin (phalloidin, green), DAPI (blue). Scale bar, 30 μm, (**C**) Number of nuclei per image for cells grown on PLGA and PGS/PLGA scaffolds quantified from confocal Z-stacks with BioLIME, (**D**) Zoomed in single XY and XZ images of nuclei stained with DAPI for cells cultured on the three substrates for 7 days. Scale bar, 2 μm. Quantification of (**E**) average nuclear width, (**F**) average nuclear height, (**G**) average monolayer height obtained with BioLIME. Data are means ± SD (*n* = 4). NS: not significant, * *p* < 0.05, ** *p* < 0.01, *** *p* < 0.001.

To determine if salivary epithelial cells grow differently on the PGS/PLGA core/shell nanofiber scaffolds than on the PLGA nanofiber scaffolds, SIMS salivary ductal epithelial cells were grown on PGS/PLGA or PLGA nanofiber scaffolds. SIMS cell growth 1 h and 1 day after culture on PGS/PLGA and PLGA nanofiber scaffolds is shown in Figure 4A. Higher absorbance values were measured in wells containing the PGS/PLGA scaffolds after 1 h, suggesting that more cells had attached onto the PGS/PLGA nanofiber scaffolds rather than the PLGA scaffolds in that time period. Furthermore, it was determined that SIMS cells on the PGS/PLGA scaffold continued to outgrow cells grown on PLGA scaffolds, showing higher absorbance readings than cells grown on PLGA scaffolds. This observation suggested that PGS/PLGA scaffolds provide an advantage to SIMS cells relative to PLGA nanofiber scaffolds for both attachment and subsequent growth. 

### 2.3. Effect of Cell Morphology on Softer PGS/PLGA Fiber Mats 

#### 2.3.1. SIMS Cell Morphology on PGS/PLGA vs. PLGA Nanofiber Substrates

Since we previously demonstrated that unmodified PLGA nanofiber scaffolds promote partial apicobasal polarization of salivary epithelial cells [11], we questioned whether PGS/PLGA nanofibers can also direct morphological changes. Confocal z-stack images were captured on different scaffolds containing SIMS cells in areas with comparable cell density (Figure 4B,C). Since we previously reported a positive correlation between cell height and nuclear height [35], we quantified nuclear morphology in cells grown on PGS/PLGA vs. PLGA scaffolds. Identified visually in zoomed in XY images (Figure 4D) and confirmed through Bio-LIME quantification, nuclear widths of cells cultured on both types of nanofibers was reduced relative to cells cultured on glass (Figure 4E). SIMS average nuclei width on glass, PLGA, and PGS/PLGA scaffold were 5.4 μm, 4.4 μm and 4.5 μm, respectively. This is likely due to the increased surface area of the nanofiber scaffolds and the decreased spreading ability of the cells when they are introduced to the nanofibrous substrates that we previously reported [34]. Confocal z-stack images, seen in zoomed in XZ images (Figure 4D), qualitatively revealed that SIMS cell nuclei cultured on the softer PGS/PLGA scaffolds were taller than cell nuclei cultured either on PLGA nanofibers or glass alone. Additionally, the average nuclear height of cells increased for the SIMS cells grown on the PGS/PLGA nanofibers relative to glass but not so for the cells grown on the PLGA nanofiber scaffolds (Figure 4F). SIMS cell average nuclear heights when cultured on glass, PLGA, and PGS/PLGA were 2.5 μm, 2.5 μm and 3.4 μm, respectively. A similar correlation for actin heights was observed on the various scaffolds showing heights of 3.4 μm, 3.5 μm, and 4.5 μm for glass, PLGA and PGS/PLGA scaffold respectively (Figure 4G). This data reveals that PGS/PLGA nanofibers modulate epithelial cell morphology more significantly than do PLGA nanofibers.

#### 2.3.2. Epithelial–Mesenchymal Cell Self-Organization and Penetration into Scaffolds 

**Figure 5:**
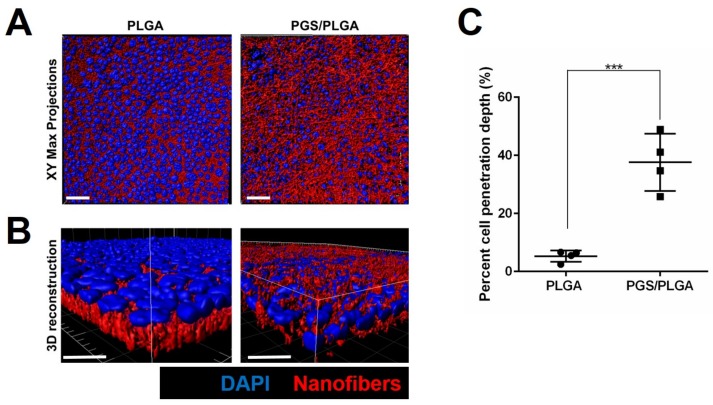
PGS/PLGA nanofibers promote cell penetration into scaffolds. (**A**,**B**) SIMS cells were cultured on PLGA or PGS/PLGA scaffolds (red) for 7 days and stained for DAPI (blue). IMARIS 3D reconstructions of Z-stacks suggest cell penetration into PGS/PLGA nanofiber mats. Scale bars, 50 μm and 10 μm for (**A**,**B**), respectively. (**C**) Quantification of cell nuclei penetration into PLGA and PGS/PLGA nanofiber scaffolds. Data are means ± SD (*n* = 4). *** *p* < 0.001 unpaired students *t*-test.

Since the PLGA nanofibers are a surface through which cells have difficulty penetrating [11,13], we examined the epithelial cell interactions with the softer PGS/PLGA scaffolds. The SIMS cell location relative to the nanofiber scaffold changed on the PGS/PLGA scaffolds when compared to the PLGA fiber mat. As expected, cells cultured on PLGA scaffolds seemed to lay on top of the nanofiber scaffold (Figure 5A,B). The cross-sectional inspection of the 3D XZ fiber mat surfaces showed deeper cell penetration within the PGS/PLGA nanofiber scaffold. Quantification of cell penetration depth revealed a significant difference between cell penetration depth on PLGA and PGS/PLGA scaffolds. The mean depth was 5.3 ± 1.9% and 33.5 ± 12.4% relative to the total scaffold depth, for PLGA and PGS/PLGA, respectively (Figure 5C), confirming an enhanced ability of the epithelial cells to penetrate the PGS/PLGA nanofibers relative to the PLGA nanofiber scaffolds.

#### 2.3.3. SIMS Cell Arrangement and Morphology with Mesenchymal Cell Interactions

**Figure 6:**
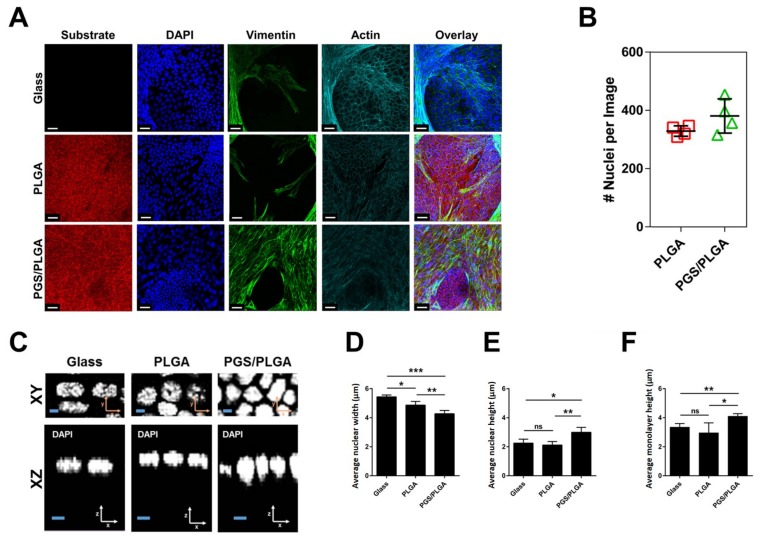
PGS/PLGA core/shell scaffolds promote enhanced epithelial self-organization. SIMS epithelial cells were grown in co-cultures with mesenchymal NIH 3T3 cells. (**A**) Maximum projection confocal images. Nanofibers (Red), nucleus (blue), actin cytoskeleton (cyan), and mesenchymal marker, vimentin (green). Scale, 30 μm, (**B**) Quantification of cell counts for SIMS cells on PLGA and PGS/PLGA scaffolds from confocal images, (**C**) Single XY and XZ zoomed in images of SIMS cell nuclei stained with DAPI when co-cultured with 3T3 cells on the three substrates for 7 days. Scale bar, 2 μm. Quantification of: (**D**) average nuclear widths, (**E**) average nuclear heights, (**F**) average monolayer heights with Bio-LIME cell. Data are means ± SD (*n* = 4). NS: not significant, * *p* < 0.05, ** *p* < 0.01, *** *p* < 0.001.

Given the enhanced ability of epithelial cells to penetrate the PGS/PLGA scaffold, we questioned if mesenchymal cells could facilitate the self-organization of epithelial cells on PGS/PLGA scaffolds. To examine epithelial morphology in the presence or absence of mesenchymal cells, SIMS epithelial cells were mixed (1:1 ratio) with NIH3T3 cells and co-cultured on PGS/PLGA vs. PLGA scaffolds in comparison with PLGA SIMS monocultures for 7 days. Immunocytochemistry was performed to detect cells with DAPI staining (blue), mesenchyme cells with vimentin (green), and cellular actin with phalloidin (cyan). Confocal Z-stacks of SIMS/3T3 co-cultures were captured for cells on each scaffold after 7 days in culture (Figure 6A). The first difference that was identified was an accumulation of mesenchymal cells surrounding epithelial cells on both substrates (Figure 6A). Z-stack top-down images were used to display this phenomenon; however, for the quantitative analysis of epithelial cell morphology, z-stack images containing only epithelial cells in the frame were analyzed. Image processing using Bio-LIME was used to quantify cell numbers and epithelial morphology differences in the same way as described in the monoculture systems. Cell counts for each confocal image indicated that there was a trend towards more epithelial cells on PGS/PLGA scaffolds than on PLGA scaffolds at this time-point (Figure 6B). 

Since Neelam et al. previously reported that nucleus shape mimics changes in cell monolayer shape [37], which we confirmed in a prior study [35], nuclear properties of epithelial cells in co-cultures and monocultures were observed in high magnification XY and XZ slices. A slight decrease in average nuclear width was detected in co-cultured epithelial cells grown on glass and PLGA scaffolds, 5.4 μm and 4.9 μm, respectively (Figure 6D). Quantification of nuclear heights showed that they remained unchanged between glass and PLGA nanofiber scaffolds in the presence of NIH3T3 cells: 2.2 μm and 2.1 μm, respectively (Figure 6E). It was qualitatively observed that SIMS nuclear heights increased on the PGS/PLGA surfaces (Figure 6C). It was also observed that co-cultured epithelium on the PGS/PLGA showed increased nuclear and monolayer heights, 3.0 μm and 4.1 μm, compared to co-cultured epithelium on PLGA scaffolds, 2.1 μm and 3.0 μm, respectively (Figure 6E,F). Epithelial monolayer heights were unchanged between glass and PLGA nanofiber scaffolds in the presence of mesenchymal cells (Figure 6F). Although the epithelial cell height increased in cells grown in co-culture with mesenchyme cells (Figure 6E) on PGS/PLGA vs. PLGA nanofibers this was not more so than in monoculture (Figure 4D). This data indicates that the contribution of mesenchymal cells to epithelial height and nuclear organization is negligible. 

### 2.4. Cell–Cell Tight Junction Localization of SIMS in Mono- and Co-Culture Systems

**Figure 7:**
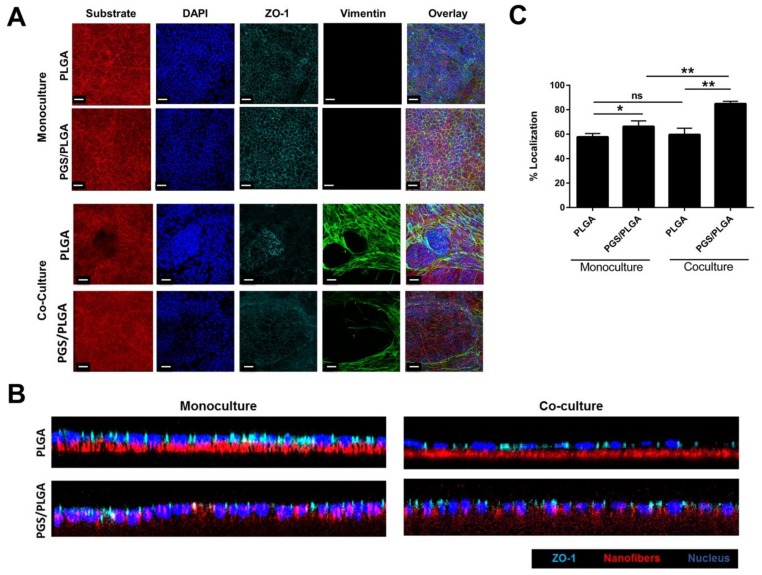
PGS/PLGA scaffolds and co-culture promote apical polarization of epithelial cells. (**A**) SIMS cells were cultured alone or co-cultured with NIH 3T3 cells on PLGA or PGS/PLGA scaffolds (red) for 7 days, stained for DAPI (blue), immunostained for vimentin (green) and ZO-1 (cyan), and confocal stacks were collected (the XY maximum projections are shown). Scale, 30 μm, (**B**) XZ slice of cells cultured on PLGA and PGS/PLGA nanofiber scaffolds. Nanofibers (red), DAPI (blue), ZO-1 tight junction protein (cyan), (**C**) Quantification of the average % apical localization of tight junction protein, ZO-1, in SIMS cells on each scaffold is shown with and without cultured NIH 3T3 cells. Data are means ± SD (*n* = 4). NS: not significant, * *p* < 0.05, ** *p* < 0.01.

Given the enhanced self-organization of epithelial cells on PGS/PLGA scaffolds vs. PLGA scaffolds, we questioned if the apicobasal polarity of the epithelial cells was increased in cells grown on the PGS/PLGA scaffolds. Confocal z-stack images were collected of SIMS cells grown alone or in the presence of NIH3T3 cells; staining of the scaffolds (red), DAPI (blue), ZO-1 tight junction protein (cyan), and vimentin (green) was performed (Figure 7A). Localization of the tight junction protein, ZO-1, was qualitatively observed in XZ slices and further quantified using Bio-LIME for both SIMS cell monoculture and co-culture (Figure 7B,C). The percent apical localization was quantified by taking each sample’s average SIMS cell nuclear height and establishing an offset that would be the top 25% and above. This was considered to be apical localization while all fluorescence below this value per sample was deemed basolateral protein expression. Z-stack images with comparable cell density were further analyzed for protein localization. Figure 7B shows X-Z cross sections of epithelial cells in monoculture and co-culture assays. It was suggested visually that cells cultured on the PGS/PLGA scaffold showed higher degrees of apical localization of the tight junction protein, ZO-1, than did cells grown on PLGA scaffolds (Figure 7B). 

Quantification of multiple images using Bio-LIME showed the SIMS cells cultured on the more compliant scaffolds, PGS/PLGA, displayed increased ZO-1 fluorescence towards the apical cell border when compared to cells grown on the PLGA nanofiber scaffolds (Figure 7C). In co-cultures, increased apical localization of ZO-1 in cells grown on PGS/PLGA vs. PLGA was more significant than in the monoculture systems, showing a *p* value of <0.01 (Figure 7C). However, when comparing the data sets of monoculture vs. co-culture no significant change of ZO-1 localization was observed between the PLGA scaffolds. This analysis indicates that epithelial apicobasal polarization is increased in cells grown on PGS/PLGA scaffolds relative to cells grown on PLGA and that the presence of mesenchymal cells further polarizes the epithelial cells. 

## 3. Materials and Methods

### 3.1. Materials

PLGA (85:15) was purchased from Durect LACTEL (Cupertino, CA, USA). Hexafluoroisopropanol (HFIP), Glycerol (reagent plus > 99% pure), and sebacic acid (99% pure) were obtained from Sigma-Aldrich (St. Louis, MO, USA). Cell Proliferation Kit 1 (MTT) was purchased from Sigma-Aldrich. Dulbecco’s phosphate buffered saline (PBS), Dulbecco’s modified Eagle’s medium (DMEM), and fetal bovine serum (FBS) were obtained from Invitrogen (Waltham, CA, USA). Sulforhodamine B (SRB) for fiber staining (Cat. No. S-1307) was purchased from Life Technologies (Grand Island, NY, USA). Vectabond Reagent was purchased from Vector Laboratories (Burlingame, CA, USA). Cell proliferation kit 1 (MTT) was from Roche (Cat. No. 11465007001; Basel, Swizerland). 4′,6-diamidino-2-phenylindole (DAPI), (for nuclei staining), and Alexafluor 488-phalloidin (to stain F-actin) were purchased from Life Technologies. Rabbit anti-Zonula occludens-1 (ZO-1) was purchased from Thermo Scientific (Cat No. 402200; Walham, MA, USA). Alexa Fluor 647-AffiniPure F(ab’)_2_ Fragment Donkey Anti-Rabbit IgG secondary antibody (Cat No. 711606152) and donkey serum were from Jackson ImmunoResearch (West Grove, PA, USA). Anti-vimentin antibody (V2258) was purchased from Sigma Aldrich (St. Louis, MO, USA), and Alexa Fluor 488 AffiniPure Donkey Anti-Mouse IgM, μchain specific (Cat No. 715545140) was purchased from Jackson ImmunoResearch. Fluor-Gel mounting media was acquired from EMS (Hatfield, PA, USA), and p-phenylenediamine (PPD) (Cat. No. P6001) was from Sigma Aldrich.

### 3.2. Synthesis of Poly (Glycerol Sebacate) (PGS)

Poly (glycerol-sebacate) (PGS) was synthesized using previously developed methods [38] which were modified, where instead of adding equimolar amounts, a 0.9:1 molar ratio of glycerol to sebacic acid was added to a round bottom flask where an overnight esterification was carried out at 120 °C. The reaction pressure was slowly reduced to 50 mTorr and the reaction continued under vacuum for 24 h resulting in PGS pre-polymer that was then used throughout this study. The band assignments to confirm nuclear magnetic resonance (^1^H-NMR) of samples before thermal curing were analyzed using a 400 MHz. The pre-polymer samples were dissolved in CDCl_3_ and the spectra was recorded at 400 MHz. The peak assignments are listed (Supplementary Figure S1). 

### 3.3. Nanofiber Fabrication

#### 3.3.1. Fabrication of Nanofibrous PLGA Scaffolds

Fabrication of PLGA nanofibers was done following previously used methods [13,34,39]. Briefly, circular glass coverslips were pre-coated with Vectabond reagent to promote polymer adhesion. Immediately prior to electrospinning, coverslips were coated with an underlayer; approximately 60 μL of 3% PLGA (*w*/*w*) dissolved in HFIP and dried at room temperature for 1 h. Using a single fluid electrospinning setup, 8% PLGA, 10 μL SRB dye and 1% salt (*w*/*w*) in HFIP was placed in a syringe pump at a flow rate of 3 μL/min. The voltage and distance of the needle from the collector plate were, 10 kV and 15 m, respectively. All samples were spun for 45 min. 

#### 3.3.2. Fabrication of Nanofibrous PGS/PLGA Scaffolds 

Circular glass coverslips were pre-coated with Vectabond reagent to promote polymer adhesion. Immediately prior to electrospinning, the coverslips were coated with an underlayer, as described for PLGA scaffolds. PGS/PLGA nanofibrous scaffolds with variable compositions were prepared using a core/shell spinneret. PGS prepolymer was dissolved in HFIP (16 wt %) and PLGA, 1% NaCl and 10 μL SRB dye was dissolved in HFIP making an 8 wt% solution. Two independent syringe pumps with variable flow rates were used with the two polymeric solutions and connected to the co-axial spinneret by PTFE tubing. The inner needle and outer needle of the co-axial spinneret were 25G (gauge) and 18G respectively (Figure 1A,B). The two solutions did not come in contact until meeting at the end of the needle tip. The same high voltage power supply was connected to both capillaries and all samples were spun for 45 min (Figure 1C).

### 3.4. Nanofiber Characterization

#### 3.4.1. Characterization of Fiber Morphology Using Scanning Electron Microscopy (SEM)

The surface topographies and fiber diameters were characterized using scanning electron microscopy (SEM) images. Samples were sputter coated with a thin layer of gold/palladium and the images were analyzed using NIH Image J software to determine fiber diameters (*n* = 100 fibers for each of the scaffolds).

#### 3.4.2. Material Characterization 

The hydrophobic/hydrophilic nature of the electrospun fibers was measured by contact angle water droplet measurements using a CAM-PLUS contact angle meter (ChemInstruments, Fairfield, OH, USA). These measurements were repeated three times on each scaffold.

The mechanical properties of polymer fiber mats were measured with atomic force microscopy (AFM) (BioScope Catalyst; Bruker, Bilerca, MA, USA). All measurements were taken in Peak Force QNM imaging mode. Polymer fiber mats were characterized using spherical borosilicate cantilevers with spring constants of 5.6 N/m, radius of 12 μm, and resonant frequency of 300 kHz (RTESPA, Bruker). Spring constant and tip radius were calibrated on a polydimethylsiloxane (PDMS) reference sample having a known Young’s Modulus of 3.5 MPa. Average Young’s modulus measurements of the fiber mats were taken from 3 separate samples from 4 different sections each. All measurements were performed on dry samples. 

### 3.5. Cell Culture

All nanofiber samples before cell seeding were sterilized by a 2 h UV treatment followed by incubation in 1X-PBS and antibiotic, 1% penicillin/streptomycin, for 24 h at 37 °C. Samples were then transferred to complete sterile media of the cell type to be incubated for another 24 h at 37 °C. For mono-culture experiments, submandibular immortalized mouse ductal salivary gland (SIMS) [39] cells were seeded 6 × 10^4^ cells/mL in 24-well plates containing nanofiber scaffolds. For co-culture experiments, NIH3T3 fibroblasts [40] and SIMS cells were trypsinized separately and combined into a centrifuge tube at a concentration of 3 × 10^4^ cells/mL each. They were mixed together and 1mL was added to each 24-plate well containing nanofiber scaffolds. SIMS and NIH3T3 fibroblast media preparation is DMEM (serum free) supplemented with final concentrations of 10% FBS and 1% 100×stock pen-strep.

### 3.6. MTT Assay

Cell growth assays were done at two time points to compare PLGA and PGS/PLGA scaffolds using cell proliferation kit 1 (MTT) with a sample set of 5 for each scaffold being tested. For both time points, 6 × 10^4^ cell/mL were added to each 24-well plate with nanofiber scaffolds at the bottom. For the binding assay, cells were allowed to attach for 1 h in the incubator. They were then washed with 1 × PBS and placed into new 24-well plates. Fresh media (DMEM, 10% FBS, 1% pen/strep) was added and 100 μL of MTT reagent 1 was added. After 4 h of incubation, MTT reagent 2 was added and left to incubate overnight. 100 μL of each samples solution was added to a 96-well plate where it was read on a plate reader. Relative absorbance was recorded at a wavelength of 570 nm with a reference wavelength of 700 nm. The same steps were followed for each time point; however, the first reagent was not added until 24 h. Wells containing only cell media were used as a baseline for absorbance measurements and were given the same amount of MTT reagents as the cell experiments. 

### 3.7. Immunocytochemistry and Confocal Microscopy

All samples for confocal microscopy were subject to immunocytochemistry (ICC) to detect cell nucleus, substrates, the tight junction protein (ZO-1), and actin. Samples, on ice for 20 min, were fixed in 4% paraformaldehyde (PFA) and 5% sucrose in 1X-PBS. Samples were washed twice in 1X-PBS-Tween (1X-PBS-T), permeabilized for 15 min in 0.1% Triton X-100 and then blocked for 2 h in 1X-PBS-T with 20% donkey serum. The primary antibody solution was incubated overnight at 4 °C on a rocker and prepared as follows; tight junction anti-rabbit (ZO-1) was added with a dilution of 1:400 in a PBS-T-3% BSA solution. Samples were washed four times/10 min with PBS-T. The secondary antibody solution was rocked at room temperature for 2 h and prepared as follows, 1:200 DAPI, 1:250 AlexFluor647 rabbit, and a 1:400 dilution of AlexFluor488-phalloidin added to a 1X-PBS-T-3%BSA solution. The samples were again washed four times/10 min each and mounted on glass slides using Fluor-gel mounting media with 1:100 p-phenylenediamine (PPD) anti-fade solution. Samples were sealed with clear nail polish and dried before imaging. Laser scanning confocal microscopy (LSCM) was performed using a Leica SP5 microscope (Leica Microscope systems, Mannheim, Germany) and images were acquired at a magnification of 63× using an oil immersion objective and 512 × 512 image resolution. Z slices of 0.5 μm in thickness were acquired for all samples. Three-dimensional reconstruction images of confocal z-stacks were constructed using IMARIS software (Bitplane, Zurich, Switzerland.

### 3.8. Statistical Analysis

Statistical analysis was carried out using an unpaired Student’s *t*-test to compare the different data sets within each experiment in GraphPad Prism 6 software (La Jolla, CA, USA). A value of *p* ≤ 0.05 was considered to be statistically significant.

### 3.9. Bio-LIME: Quantitative Analysis for Cell Penetration Depth, Cell Morphology and Cell–Cell Junction Protein Localization Measurements

Bio-LIME (bio-image matrix evaluation) was developed to quantify morphological features of cells cultured on scaffolds from z-stacks of confocal images [35]. All confocal channels were separately exported as .tiff images and processed through the program. Using the nanofiber channel to define the base of the scaffold, all the other channels were overlaid on this channel. Cell penetration depths were determined as follows: the average distance between the scaffold base and cell nucleus base was divided by the overall height of the scaffold and multiplied by 100. Nuclear heights and widths were analyzed by counting the continuous path of fluorescence in pixels of the DAPI channel (cell nuclei). Average cell monolayer heights were determined using the top most actin fluorescence stain and the bottom-most DAPI fluorescence. The percent localization of ZO-1 protein was determined by setting an offset position at 25% and above the total height of the average nuclear height. This offset was established keeping in mind that fluorescence can be distorted slightly due to photo bleaching and poor resolution in the z dimension. The protein below this offset is defined as the amount of basolateral fluorescence while protein observed above the top 25% is defined as apical protein localization. Four confocal Z-stacks were acquired for each scaffold and quantified through Bio-LIME. 

## 4. Discussion

The ability of cells to self-organize is a critical requirement for tissue regeneration. Although they are able to deliver topological and chemical signals to cells, epithelial cells grown on PLGA-based nanofiber scaffolds allow relatively little cell self-organization. Salivary epithelial cells cultured on PGS/PLGA fiber mats penetrated through the mat rather than just laying on top, as seen on PLGA scaffolds. This observation of cells integrating themselves into the more compliant nanofiber mat suggests that the cells would be better able to self-organize within this environment. In fact, the salivary epithelial cells responded to the PGS/PLGA scaffolds differently than the PLGA scaffolds, yielding a more in vivo-like morphology and tight junction proteins localized towards the apical side of cell monolayers. Incorporating PGS into PLGA nanofiber scaffolds yields a minor increase in the compliance of the scaffolds and also increases the hydrophilic nature of the scaffold, which is known to aid in cell attachment. The cell growth results showed SIMS cell adhesion to be significantly higher on the PGS/PLGA scaffolds compared to PLGA scaffolds in a short-term measurement. Increased hydrophilicity can be due to the interaction that PLGA and PGS have with one another resulting in increased surface roughness and pores within the fiber. Increased hydrophilicity of scaffolds has been shown to enhance the overall absorption and diffusion of culture medium which in turn increases the ability of cells to both penetrate the scaffolds and to attach to them [21]. The increased compliance and/or the hydrophilic nature of the PGS/PLGA scaffolds may contribute to the increased ability of cells to penetrate the fiber mat and grow within it relative to PLGA nanofibers. 

Nucleus shape mimicking changes in cell monolayer structure described by Neelam et al. [37] and nuclear heights correlating with increasing monolayer height reported by Li et al. may be due to the physical connection of the plasma membrane to the nuclear membrane that is accomplished through the mechanical coupling of the cytoskeleton with the nuclear matrix [41]. Here, nuclear morphology also followed plasma membrane expansion or compression in response to the substrate, as in our prior work [35].

## 5. Conclusions

The incorporation of PGS into PLGA nanofiber scaffolds using a core/shell eletrospinning technique affected the morphology and apicobasal polarization of salivary epithelial cells, as determined by the apical localization of the tight junction protein, ZO-1. We demonstrated increased cell binding of salivary epithelial cells on the PGS/PLGA nanofibers compared to PLGA nanofibers. Increased nucleus and cell monolayer heights of epithelial cells grown on PGS/PLGA scaffolds were enhanced by co-culture with mesenchymal cells. Increased apical polarization of the tight junction protein, ZO-1, was also achieved with the PGS/PLGA nanofibers relative to the PLGA nanofibers, which was further enhanced by co-culture with NIH3T3 mesenchymal cells. 

Co-culturing epithelial cells with mesenchymal cells revealed that the epithelial cell organization was more in vivo like when cells were cultured on the PGS/PLGA scaffolds, which allow more penetration into the scaffold and cell self-organization than do PLGA scaffolds. In our study, significant increases in tight junction protein apical localization were observed in epithelial cells cultured on PGS/PLGA scaffolds when the epithelial cells interacted with the mesenchymal cell population and less so without them. These results suggest the importance of epithelial-mesenchymal cell interactions in the early stages of in vitro tissue formation, which will translate to more effective tissue assembly in vivo for future regenerative therapies. 

## Figures and Tables

**Table 1:** Properties of PLGA and PGS/PLGA nanofiber scaffolds.

Scaffold Type	Young’s Modulus (MPa)	Water Contact Angle (Mean ± Stdev)
PLGA	4.8 ± 1.5	122 ± 4
PGS/PLGA	1.3 ± 0.66	65 ± 5

## Supplementary Materials

Supplementary materials can be found at http://www.mdpi.com/1422-0067/19/4/1031/s1.
