# Supplementary material for: Mesenchymal Cells Affect Salivary Epithelial Cell Morphology on PGS/PLGA Core/Shell Nanofibers
Source: Int J Mol Sci. 2018 Mar 29;19(4):1031. doi: 10.3390/ijms19041031 (PMC5979364; doi:10.3390/ijms19041031)
Supplement: Supplementary file 1 [file ijms-19-01031-s001.pdf]

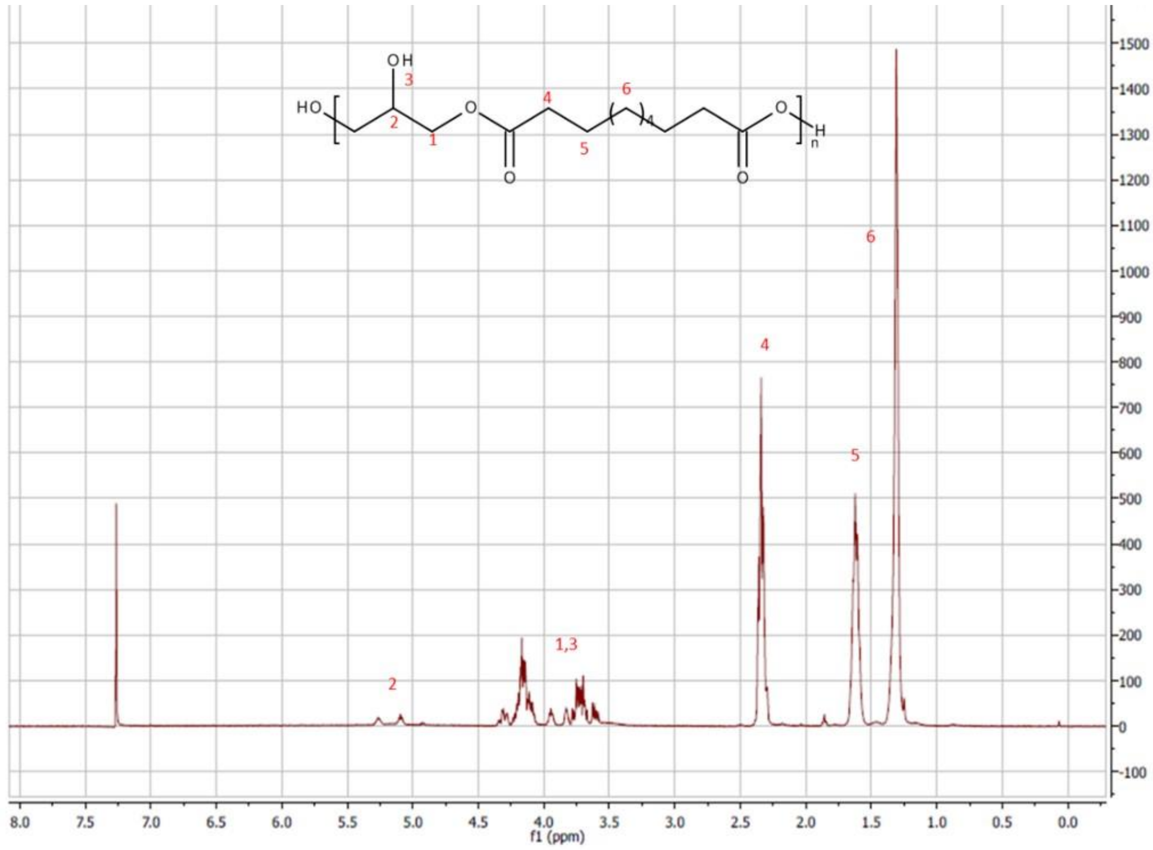


**Supplemental Figure S1.** Measured NMR spectrum of 0.9:1 PGS prepolymer. $^{1}$H NMR **(0.9:1 PGS**) (400 MHz, CDCl3) d/ppm: 1.31 (m, -CH2), 1.61 (d, -CH2CH2O(CO)-), 2.34 (m,-CH2O(CO)), 3.61-3.82 (m, OHCH2CHO), 3.93 (m,-OCH2CHOH, 4.05-3.35 (m, -OCH2CHO, 5.11 (s, OHCH2CHO), 5.27 (s, -OCH2CHO)
